# Ethyl 7′-(6-benz­yloxy-2,2-dimethyl­tetra­hydro­furo[3,2-*d*][1,3]dioxol-5-yl)-2-oxo-5′,6′,7′,7a’-tetra­hydro-1′*H*,2*H*-spiro­[acenaphthyl­ene-1,5′-pyrrolo­[1,2-*c*][1,3]thia­zole]-6′-carboxyl­ate

**DOI:** 10.1107/S1600536812032291

**Published:** 2012-07-21

**Authors:** G. Jagadeesan, K. Sethusankar, R. Prasanna, R. Raghunathan

**Affiliations:** a Department of Physics, Dr MGR Educational and Research Institute, Dr MGR University, Chennai 600 095, India; b Department of Physics, RKM Vivekananda College (Autonomous), Chennai 600 004, India; c Department of Organic Chemistry, University of Madras, Maraimalai Campus, Chennai 600 025, India

## Abstract

In the title compound, C_34_H_35_NO_7_S, the acenaphthyl­ene unit is essentially planar (r.m.s. deviation = 0.0335 Å). The pyrrolo­thia­zole ring system is folded about the bridging N—C bond; the thia­zolidine and pyrrolidine rings adopt S- and C-envelope conformations, respectively, with a ‘butterfly’ angle between the mean planes of 51.38 (10)°. The dioxolane and tetra­hydro­furan rings adopt O- and a C-envelope conformations, respectively, with a ‘butterfly’ angle between the mean planes of 57.12 (10)°. Two C atoms are each disordered over two positions with site-occupancy factors of 0.450 (7) and 0.550 (7). The crystal packing is stabilized by C—H⋯O inter­actions, generating an *R*_2_^2^(14) graph-set ring motif.

## Related literature

For the biological properties of spiro­heterocycles, see: Kilonda *et al.* (1995 ▶); Ferguson *et al.* (2005 ▶). For a related structure, see: Jagadeesan *et al.* (2012 ▶). For graph-set notation, see: Bernstein *et al.* (1995 ▶).
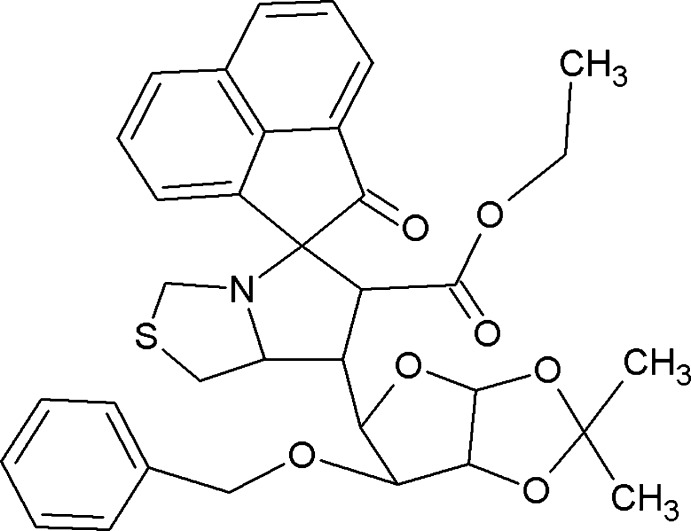


## Experimental

### 

#### Crystal data

C_34_H_35_NO_7_S
*M* *_r_* = 601.69
Monoclinic, 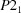

*a* = 8.588 (5) Å
*b* = 20.446 (5) Å
*c* = 8.851 (5) Å
β = 93.282 (5)°
*V* = 1551.6 (13) Å^3^
*Z* = 2
Mo *K*α radiation
μ = 0.15 mm^−1^
*T* = 293 K
0.30 × 0.20 × 0.20 mm

#### Data collection

Bruker Kappa APEXII CCD diffractometer
Absorption correction: multi-scan (*SADABS*; Sheldrick, 1996 ▶) *T*_min_ = 0.955, *T*_max_ = 0.970
13521 measured reflections
5470 independent reflections
4678 reflections with *I* > 2σ(*I*)
*R*_int_ = 0.031

#### Refinement

*R*[*F* ^2^ > 2σ(*F* ^2^)] = 0.037
*wR*(*F* ^2^) = 0.083
*S* = 1.03
5470 reflections
400 parameters
5 restraints
H-atom parameters constrained
Δρ_max_ = 0.17 e Å^−3^
Δρ_min_ = −0.16 e Å^−3^
Absolute structure: Flack (1983 ▶), 2630 Friedel pairs
Flack parameter: 0.04 (7)

### 

Data collection: *APEX2* (Bruker, 2008 ▶); cell refinement: *SAINT* (Bruker, 2008 ▶); data reduction: *SAINT*; program(s) used to solve structure: *SHELXS97* (Sheldrick, 2008 ▶); program(s) used to refine structure: *SHELXL97* (Sheldrick, 2008 ▶); molecular graphics: *ORTEP-3* (Farrugia, 1997 ▶); software used to prepare material for publication: *SHELXL97* and *PLATON* (Spek, 2009 ▶).

## Supplementary Material

Crystal structure: contains datablock(s) global, I. DOI: 10.1107/S1600536812032291/pv2565sup1.cif

Structure factors: contains datablock(s) I. DOI: 10.1107/S1600536812032291/pv2565Isup2.hkl

Supplementary material file. DOI: 10.1107/S1600536812032291/pv2565Isup3.cml

Additional supplementary materials:  crystallographic information; 3D view; checkCIF report

## Figures and Tables

**Table 1:** Hydrogen-bond geometry (Å, °)

*D*—H⋯*A*	*D*—H	H⋯*A*	*D*⋯*A*	*D*—H⋯*A*
C5—H5⋯O3^i^	0.93	2.51	3.375 (4)	155
C17—H17*A*⋯O7^ii^	0.97	2.44	3.309 (3)	148
C23—H23*F*⋯O4^iii^	0.96	2.57	3.497 (9)	163
C31—H31⋯O5^iv^	0.93	2.47	3.393 (4)	173

Symmetry codes: (i) 
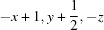
; (ii) 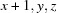; (iii) 
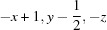
; (iv) 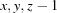.

**Fig. 1.**
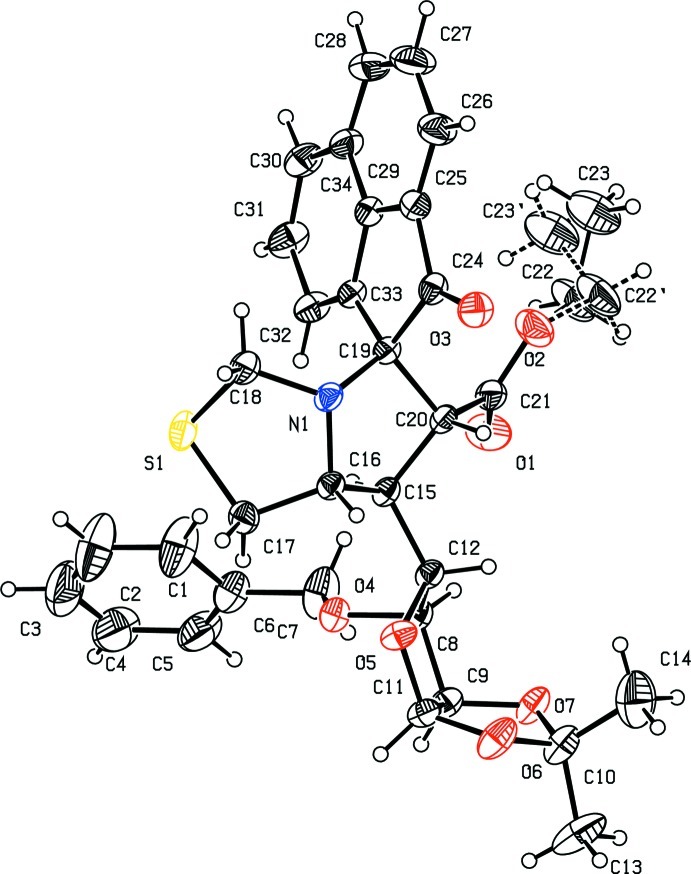
The molecular structure of the title compound with the atom numbering scheme, displacement ellipsoids are drawn at 30% probability level. H–atoms are present as small spheres of arbitary radius. The minor fractions of the disordered carbon atoms have been represented by broken bonds.

**Fig. 2.**
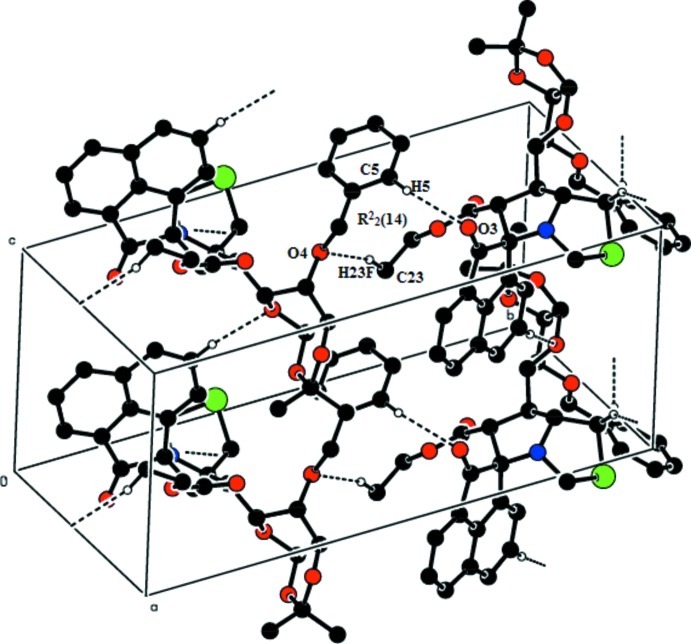
The crystal packing of the title compound viewed down c axis, showing the hydrogen bonds resulting in R22(14) graph-set ring motif. H–atoms not involved in hydrogen bonds have been excluded for clarity.

## supplementary crystallographic information

### Comment

The design and novel synthesis of glycospiroheterocycles are interesting because of the synthetic challenge they present and their biological profile against viruses, bacteria, and cancer cells (Ferguson *et al.*, 2005). Pyrrolidines and pyrroles are common structural motifs in drugs and drug candidates owing to their ability to act as selective glycosidase inhibitors, which are used in the treatment of diabetes, cancer, malaria and viral infections, including AIDS (Kilonda *et al.*, 1995).

In the title molecule (Fig. 1), the acenaphthylene moiety (C19/C24–C34) is essentially planar (rmsd = 0.0335 Å) with O3 deviating from the acenaphthylene moiety by 0.209 (3) Å. The pyrrolothiazole ring (C15–C20/N1/S1) system is folded about the bridging N1–C16 bond, as observed in another structurally similar compound (Jagadeesan *et al.*, 2012). The thiazolidine ring (C16–C18/N1/S1) adopts an S1-*envelope* conformation with S1 deviating from the mean plane of the remaining ring atoms by 0.815 (3) Å while the pyrrolidine ring (C15/C16/C19/C20/N1) adopt adopts a C20-*envelope* conformation with C20 deviating from the mean plane of the remaining ring atoms by 0.538 (3) Å; the "butter-fly" angle between the mean planes C16-C18/N1 and C15/C16/C19/N1 being 51.38 (10) °. The dioxolane ring (C9–C11/O6/O7) adopts an O7-*envelope* conformation with the atom O7 deviating from the mean plane of the remaining ring atoms by 0.299 (4) Å. The tetrahydrofuran ring (O5/C8/C9/C11/C12) adopts a C12-*envelope* conformation with C12 deviating from the mean plane of the remaining ring atoms by 0.607 (3) Å; the "butter-fly" angle between the mean planes O5/C8/C9/C11 and O6/C9–C11 is 57.12 (10) °.

The crystal packing is stabilized by C—H···O intermolecular interactions; C5—H5···O3 and C23—H23F···O4 hydrogen bonds generate *R*^2^_2_(14) graphset ring motif (Bernstein, *et al.*, 1995) (Table 1 and Fig. 2).

### Experimental

A mixture of α-*D*-*xylo*-hept-5-enofuranuronic acid, 5,6-dideoxy-1,2-*O*-(1-methylethylidene)-3-*O*- (phenylmethyl)-, ethyl ester (0.300 g, 0.86 mmol), acenaphthenequinone (0.156 g, 0.86 mmol) and 4-thiazolidinecarboxyli cacid (0.137 g, 1.0 mmol) was refluxed in toluene for about 5 h under Dean stark reaction condition to yield the title compound. After the completion of reaction as indicated by TLC, solvent was evaporated under reduced pressure. The crude product was purified by column chromatography using hexane: EtOAc (4:1) as eluent. The block shaped single crystals of the title compound suitable for X-ray diffraction were obtained from solution of hexane: EtOAc (4:1) by slow evaportion at room temperature.

### Refinement

An absolute structure was determined by the Flack method (Flack, 1983) using 2630 Friedel pairs of reflections which were not merged. The hydrogen atoms were placed in calculated positions with C–H = 0.93 - 0.97 Å refined in the riding model with fixed isotropic displacement parameters: *U*_iso_(H) = 1.5 *U*_eq_(C) for methyl group and *U*_iso_(H) = 1.2 *U*_eq_(C) for other groups. The bond distances of the disordered components were restrained using standard similarity restraint SADI [*SHELXL97*, Sheldrick, 2008] with s.u. of 0.01 Å. The atomic displacement parameter of the major and minor components were made similar using the constraint EADP. The rigid bond restraint DELU is applied between the disordered atoms C22, C23 and C22', C23' with s.u. of 0.01.

### Crystal data

**Table:**

C_34_H_35_NO_7_S	*F*(000) = 636
*M**_r_* = 601.69	*D*_x_ = 1.288 Mg m^−^^3^
Monoclinic, *P*2_1_	Mo *K*α radiation, λ = 0.71073 Å
Hall symbol: P 2yb	Cell parameters from 5470 reflections
*a* = 8.588 (5) Å	θ = 2.3–25.0°
*b* = 20.446 (5) Å	µ = 0.15 mm^−^^1^
*c* = 8.851 (5) Å	*T* = 293 K
β = 93.282 (5)°	Block, colourless
*V* = 1551.6 (13) Å^3^	0.30 × 0.20 × 0.20 mm
*Z* = 2	

### Data collection

**Table:**

Bruker Kappa APEXII CCD diffractometer	5470 independent reflections
Radiation source: fine-focus sealed tube	4678 reflections with *I* > 2σ(*I*)
Graphite monochromator	*R*_int_ = 0.031
ω scans	θ_max_ = 25.0°, θ_min_ = 2.3°
Absorption correction: multi-scan (*SADABS*; Sheldrick, 1996)	*h* = −9→10
*T*_min_ = 0.955, *T*_max_ = 0.970	*k* = −24→24
13521 measured reflections	*l* = −10→10

### Refinement

**Table:**

Refinement on *F*^2^	Secondary atom site location: difference Fourier map
Least-squares matrix: full	Hydrogen site location: inferred from neighbouring sites
*R*[*F*^2^ > 2σ(*F*^2^)] = 0.037	H-atom parameters constrained
*wR*(*F*^2^) = 0.083	*w* = 1/[σ^2^(*F*_o_^2^) + (0.0365*P*)^2^ + 0.1903*P*] where *P* = (*F*_o_^2^ + 2*F*_c_^2^)/3
*S* = 1.03	(Δ/σ)_max_ = 0.001
5470 reflections	Δρ_max_ = 0.17 e Å^−^^3^
400 parameters	Δρ_min_ = −0.16 e Å^−^^3^
5 restraints	Absolute structure: Flack (1983), 2630 Friedel pairs
Primary atom site location: structure-invariant direct methods	Flack parameter: 0.04 (7)

### Special details

**Table:**

Geometry. All e.s.d.'s (except the e.s.d. in the dihedral angle between two l.s. planes) are estimated using the full covariance matrix. The cell e.s.d.'s are taken into account individually in the estimation of e.s.d.'s in distances, angles and torsion angles; correlations between e.s.d.'s in cell parameters are only used when they are defined by crystal symmetry. An approximate (isotropic) treatment of cell e.s.d.'s is used for estimating e.s.d.'s involving l.s. planes.
Refinement. Refinement of *F*^2^ against ALL reflections. The weighted *R*-factor *wR* and goodness of fit *S* are based on *F*^2^, conventional *R*-factors *R* are based on *F*, with *F* set to zero for negative *F*^2^. The threshold expression of *F*^2^ > σ(*F*^2^) is used only for calculating *R*-factors(gt) *etc*. and is not relevant to the choice of reflections for refinement. *R*-factors based on *F*^2^ are statistically about twice as large as those based on *F*, and *R*- factors based on ALL data will be even larger.

### Fractional atomic coordinates and isotropic or equivalent isotropic displacement parameters (Å^2^)

**Table:**

	*x*	*y*	*z*	*U*_iso_*/*U*_eq_	Occ. (<1)
C1	0.6985 (4)	1.0180 (2)	−0.1860 (4)	0.0964 (12)	
H1	0.7439	0.9791	−0.1517	0.116*	
C2	0.7881 (4)	1.0639 (3)	−0.2530 (5)	0.1233 (17)	
H2	0.8946	1.0569	−0.2592	0.148*	
C3	0.7226 (6)	1.1190 (2)	−0.3098 (5)	0.1204 (15)	
H3	0.7837	1.1503	−0.3544	0.145*	
C4	0.5673 (7)	1.12883 (19)	−0.3019 (5)	0.1210 (16)	
H4	0.5213	1.1662	−0.3442	0.145*	
C5	0.4768 (4)	1.08343 (17)	−0.2310 (4)	0.0861 (10)	
H5	0.3703	1.0906	−0.2259	0.103*	
C6	0.5424 (3)	1.02855 (15)	−0.1686 (3)	0.0598 (7)	
C7	0.4478 (3)	0.97918 (18)	−0.0920 (3)	0.0767 (9)	
H7A	0.3442	0.9965	−0.0788	0.092*	
H7B	0.4373	0.9401	−0.1540	0.092*	
C8	0.4389 (2)	0.91510 (11)	0.1311 (2)	0.0385 (5)	
H8	0.3568	0.8941	0.0667	0.046*	
C9	0.3749 (2)	0.94455 (12)	0.2722 (2)	0.0444 (5)	
H9	0.3382	0.9896	0.2576	0.053*	
C10	0.2888 (3)	0.89133 (16)	0.4821 (2)	0.0579 (7)	
C11	0.5109 (2)	0.93842 (12)	0.3919 (2)	0.0448 (5)	
H11	0.5512	0.9815	0.4227	0.054*	
C12	0.5534 (2)	0.86545 (11)	0.2003 (2)	0.0371 (5)	
H12	0.4946	0.8287	0.2394	0.045*	
C13	0.1895 (4)	0.9367 (2)	0.5694 (3)	0.0944 (12)	
H13A	0.2072	0.9285	0.6758	0.142*	
H13B	0.0816	0.9295	0.5401	0.142*	
H13C	0.2165	0.9812	0.5481	0.142*	
C14	0.2592 (5)	0.8205 (2)	0.5147 (5)	0.1105 (13)	
H14A	0.3315	0.7940	0.4630	0.166*	
H14B	0.1546	0.8094	0.4802	0.166*	
H14C	0.2727	0.8129	0.6217	0.166*	
C15	0.6747 (2)	0.83868 (11)	0.0969 (2)	0.0348 (5)	
H15	0.6642	0.8626	0.0009	0.042*	
C16	0.8441 (2)	0.84394 (11)	0.1576 (2)	0.0358 (5)	
H16	0.8497	0.8358	0.2669	0.043*	
C17	0.9220 (2)	0.90903 (12)	0.1268 (3)	0.0475 (6)	
H17A	0.9987	0.9196	0.2078	0.057*	
H17B	0.8451	0.9438	0.1190	0.057*	
C18	1.0597 (3)	0.81554 (12)	0.0079 (3)	0.0522 (6)	
H18A	1.0795	0.7891	−0.0799	0.063*	
H18B	1.1515	0.8145	0.0770	0.063*	
C19	0.8148 (2)	0.74831 (11)	−0.0048 (2)	0.0346 (5)	
C20	0.6583 (2)	0.76627 (10)	0.0622 (2)	0.0361 (5)	
H20	0.6520	0.7429	0.1583	0.043*	
C21	0.5169 (3)	0.74830 (13)	−0.0381 (3)	0.0469 (6)	
C24	0.8642 (2)	0.67578 (11)	0.0284 (2)	0.0402 (5)	
C25	0.9027 (3)	0.64571 (11)	−0.1152 (3)	0.0447 (5)	
C26	0.9563 (3)	0.58507 (13)	−0.1508 (3)	0.0633 (7)	
H26	0.9807	0.5543	−0.0758	0.076*	
C27	0.9734 (4)	0.57065 (14)	−0.3031 (3)	0.0784 (9)	
H27	1.0113	0.5297	−0.3286	0.094*	
C28	0.9366 (4)	0.61442 (15)	−0.4160 (3)	0.0701 (8)	
H28	0.9489	0.6026	−0.5160	0.084*	
C29	0.8805 (3)	0.67706 (13)	−0.3835 (3)	0.0501 (6)	
C30	0.8345 (3)	0.72664 (15)	−0.4870 (3)	0.0603 (7)	
H30	0.8428	0.7201	−0.5902	0.072*	
C31	0.7775 (3)	0.78419 (14)	−0.4354 (3)	0.0606 (7)	
H31	0.7448	0.8160	−0.5054	0.073*	
C32	0.7663 (3)	0.79738 (13)	−0.2803 (2)	0.0505 (6)	
H32	0.7273	0.8373	−0.2490	0.061*	
C33	0.8127 (2)	0.75142 (11)	−0.1771 (2)	0.0371 (5)	
C34	0.8683 (2)	0.69111 (11)	−0.2306 (2)	0.0411 (5)	
N1	0.92549 (18)	0.79087 (9)	0.08164 (18)	0.0381 (4)	
O1	0.4185 (2)	0.78642 (10)	−0.0821 (2)	0.0725 (6)	
O3	0.8674 (2)	0.65153 (8)	0.15251 (18)	0.0547 (4)	
O4	0.52013 (16)	0.96312 (8)	0.05080 (16)	0.0437 (4)	
O5	0.62621 (14)	0.90082 (8)	0.32626 (14)	0.0443 (4)	
O6	0.44787 (17)	0.90588 (11)	0.51319 (16)	0.0641 (5)	
O7	0.26019 (15)	0.90235 (10)	0.32494 (15)	0.0540 (4)	
S1	1.01420 (7)	0.89962 (4)	−0.04894 (7)	0.05884 (19)	
O2	0.5175 (2)	0.68557 (9)	−0.0717 (2)	0.0714 (5)	
C22'	0.3864 (13)	0.6486 (8)	−0.1444 (12)	0.086 (3)	0.450 (7)
H22A	0.2876	0.6703	−0.1319	0.103*	0.450 (7)
H22B	0.3818	0.6044	−0.1052	0.103*	0.450 (7)
C23'	0.4283 (13)	0.6493 (6)	−0.3035 (11)	0.1009 (19)	0.450 (7)
H23A	0.4392	0.6937	−0.3366	0.151*	0.450 (7)
H23B	0.3478	0.6281	−0.3653	0.151*	0.450 (7)
H23C	0.5251	0.6265	−0.3124	0.151*	0.450 (7)
C22	0.3883 (11)	0.6700 (5)	−0.1829 (12)	0.086 (3)	0.550 (7)
H22C	0.2916	0.6651	−0.1326	0.103*	0.550 (7)
H22D	0.3754	0.7047	−0.2572	0.103*	0.550 (7)
C23	0.4290 (11)	0.6083 (4)	−0.2567 (10)	0.1009 (19)	0.550 (7)
H23D	0.5275	0.6131	−0.3016	0.151*	0.550 (7)
H23E	0.3499	0.5977	−0.3338	0.151*	0.550 (7)
H23F	0.4362	0.5739	−0.1828	0.151*	0.550 (7)

### Atomic displacement parameters (Å^2^)

**Table:**

	*U*^11^	*U*^22^	*U*^33^	*U*^12^	*U*^13^	*U*^23^
C1	0.0573 (19)	0.134 (3)	0.098 (2)	0.0049 (18)	0.0028 (16)	0.065 (2)
C2	0.079 (2)	0.175 (5)	0.117 (3)	−0.009 (3)	0.015 (2)	0.080 (3)
C3	0.129 (4)	0.115 (4)	0.122 (3)	−0.018 (3)	0.051 (3)	0.036 (3)
C4	0.175 (5)	0.071 (2)	0.125 (3)	0.031 (3)	0.077 (3)	0.037 (2)
C5	0.092 (2)	0.084 (2)	0.087 (2)	0.0304 (19)	0.0397 (19)	0.0205 (19)
C6	0.0566 (15)	0.079 (2)	0.0437 (14)	0.0024 (14)	0.0009 (11)	0.0184 (14)
C7	0.0585 (17)	0.111 (3)	0.0592 (17)	−0.0106 (17)	−0.0081 (13)	0.0336 (17)
C8	0.0280 (9)	0.0504 (15)	0.0372 (11)	−0.0020 (9)	0.0039 (8)	0.0018 (10)
C9	0.0380 (12)	0.0514 (14)	0.0445 (13)	0.0096 (10)	0.0072 (10)	0.0016 (11)
C10	0.0417 (12)	0.090 (2)	0.0424 (12)	0.0033 (14)	0.0073 (10)	0.0081 (14)
C11	0.0417 (12)	0.0520 (14)	0.0411 (12)	0.0047 (10)	0.0071 (10)	−0.0068 (11)
C12	0.0296 (10)	0.0476 (14)	0.0345 (11)	−0.0010 (9)	0.0036 (9)	−0.0028 (9)
C13	0.0661 (18)	0.170 (4)	0.0487 (16)	0.029 (2)	0.0149 (14)	−0.0141 (19)
C14	0.119 (3)	0.109 (3)	0.105 (3)	−0.024 (2)	0.013 (2)	0.036 (2)
C15	0.0286 (10)	0.0455 (13)	0.0305 (10)	0.0004 (9)	0.0021 (8)	0.0014 (9)
C16	0.0313 (10)	0.0418 (12)	0.0345 (11)	−0.0003 (9)	0.0028 (9)	−0.0012 (9)
C17	0.0340 (10)	0.0476 (15)	0.0608 (14)	−0.0032 (11)	0.0013 (9)	−0.0043 (12)
C18	0.0345 (12)	0.0582 (16)	0.0648 (15)	−0.0015 (11)	0.0107 (10)	−0.0054 (13)
C19	0.0321 (10)	0.0377 (11)	0.0346 (11)	0.0023 (9)	0.0052 (8)	0.0009 (9)
C20	0.0321 (10)	0.0452 (13)	0.0317 (11)	−0.0022 (9)	0.0065 (8)	−0.0009 (9)
C21	0.0345 (12)	0.0538 (15)	0.0527 (14)	−0.0048 (11)	0.0055 (10)	−0.0127 (12)
C24	0.0371 (11)	0.0436 (13)	0.0401 (12)	0.0022 (10)	0.0035 (9)	0.0069 (10)
C25	0.0463 (13)	0.0412 (13)	0.0468 (13)	0.0013 (10)	0.0054 (10)	0.0007 (10)
C26	0.089 (2)	0.0420 (15)	0.0600 (17)	0.0096 (14)	0.0137 (14)	0.0007 (12)
C27	0.117 (2)	0.0443 (16)	0.076 (2)	0.0183 (17)	0.0216 (18)	−0.0119 (15)
C28	0.094 (2)	0.0634 (19)	0.0549 (16)	0.0003 (16)	0.0226 (15)	−0.0207 (15)
C29	0.0542 (14)	0.0567 (16)	0.0404 (13)	−0.0010 (12)	0.0104 (10)	−0.0077 (12)
C30	0.0706 (17)	0.078 (2)	0.0330 (13)	0.0014 (15)	0.0098 (12)	−0.0029 (13)
C31	0.0716 (17)	0.0724 (19)	0.0383 (13)	0.0173 (15)	0.0068 (11)	0.0142 (13)
C32	0.0549 (14)	0.0560 (16)	0.0416 (13)	0.0129 (12)	0.0100 (10)	0.0027 (12)
C33	0.0342 (11)	0.0409 (12)	0.0367 (11)	0.0032 (9)	0.0061 (8)	0.0009 (10)
C34	0.0391 (11)	0.0434 (14)	0.0414 (12)	−0.0023 (10)	0.0073 (9)	−0.0011 (10)
N1	0.0274 (8)	0.0453 (11)	0.0420 (10)	0.0013 (8)	0.0058 (7)	−0.0026 (9)
O1	0.0433 (10)	0.0815 (14)	0.0902 (14)	0.0097 (10)	−0.0177 (9)	−0.0275 (12)
O3	0.0681 (11)	0.0518 (10)	0.0443 (10)	0.0055 (8)	0.0057 (8)	0.0112 (8)
O4	0.0403 (8)	0.0511 (10)	0.0394 (8)	−0.0005 (7)	0.0001 (6)	0.0072 (7)
O5	0.0342 (7)	0.0624 (10)	0.0358 (7)	0.0096 (8)	−0.0017 (6)	−0.0075 (8)
O6	0.0459 (9)	0.1101 (15)	0.0364 (8)	0.0056 (10)	0.0033 (6)	0.0089 (10)
O7	0.0340 (7)	0.0880 (12)	0.0405 (8)	−0.0025 (9)	0.0080 (6)	0.0014 (9)
S1	0.0471 (3)	0.0637 (4)	0.0672 (4)	−0.0115 (3)	0.0167 (3)	0.0101 (4)
O2	0.0556 (11)	0.0612 (13)	0.0963 (14)	−0.0141 (9)	−0.0061 (10)	−0.0252 (11)
C22'	0.079 (2)	0.057 (7)	0.119 (5)	−0.017 (3)	−0.024 (3)	−0.027 (5)
C23'	0.121 (4)	0.081 (5)	0.096 (4)	0.002 (5)	−0.032 (4)	−0.024 (4)
C22	0.079 (2)	0.057 (7)	0.119 (5)	−0.017 (3)	−0.024 (3)	−0.027 (5)
C23	0.121 (4)	0.081 (5)	0.096 (4)	0.002 (5)	−0.032 (4)	−0.024 (4)

### Geometric parameters (Å, º)

**Table:**

C1—C2	1.370 (5)	C17—H17B	0.9700
C1—C6	1.375 (4)	C18—N1	1.447 (3)
C1—H1	0.9300	C18—S1	1.827 (3)
C2—C3	1.344 (6)	C18—H18A	0.9700
C2—H2	0.9300	C18—H18B	0.9700
C3—C4	1.354 (6)	C19—N1	1.471 (3)
C3—H3	0.9300	C19—C33	1.525 (3)
C4—C5	1.384 (5)	C19—C20	1.544 (3)
C4—H4	0.9300	C19—C24	1.566 (3)
C5—C6	1.359 (4)	C20—C21	1.508 (3)
C5—H5	0.9300	C20—H20	0.9800
C6—C7	1.484 (4)	C21—O1	1.198 (3)
C7—O4	1.415 (3)	C21—O2	1.317 (3)
C7—H7A	0.9700	C24—O3	1.204 (3)
C7—H7B	0.9700	C24—C25	1.466 (3)
C8—O4	1.419 (3)	C25—C26	1.366 (3)
C8—C9	1.517 (3)	C25—C34	1.399 (3)
C8—C12	1.518 (3)	C26—C27	1.396 (4)
C8—H8	0.9800	C26—H26	0.9300
C9—O7	1.409 (3)	C27—C28	1.365 (4)
C9—C11	1.537 (3)	C27—H27	0.9300
C9—H9	0.9800	C28—C29	1.404 (4)
C10—O6	1.410 (3)	C28—H28	0.9300
C10—O7	1.417 (3)	C29—C34	1.393 (3)
C10—C14	1.501 (4)	C29—C30	1.408 (4)
C10—C13	1.503 (4)	C30—C31	1.363 (4)
C11—O6	1.398 (3)	C30—H30	0.9300
C11—O5	1.406 (3)	C31—C32	1.408 (3)
C11—H11	0.9800	C31—H31	0.9300
C12—O5	1.441 (2)	C32—C33	1.355 (3)
C12—C15	1.526 (3)	C32—H32	0.9300
C12—H12	0.9800	C33—C34	1.414 (3)
C13—H13A	0.9600	O2—C22'	1.473 (7)
C13—H13B	0.9600	O2—C22	1.475 (6)
C13—H13C	0.9600	C22'—C23'	1.474 (10)
C14—H14A	0.9600	C22'—H22A	0.9700
C14—H14B	0.9600	C22'—H22B	0.9700
C14—H14C	0.9600	C23'—H23A	0.9600
C15—C20	1.517 (3)	C23'—H23B	0.9600
C15—C16	1.526 (3)	C23'—H23C	0.9600
C15—H15	0.9800	C22—C23	1.471 (9)
C16—N1	1.474 (3)	C22—H22C	0.9700
C16—C17	1.521 (3)	C22—H22D	0.9700
C16—H16	0.9800	C23—H23D	0.9600
C17—S1	1.795 (2)	C23—H23E	0.9600
C17—H17A	0.9700	C23—H23F	0.9600
			
C2—C1—C6	121.1 (4)	H17A—C17—H17B	108.6
C2—C1—H1	119.4	N1—C18—S1	106.80 (15)
C6—C1—H1	119.4	N1—C18—H18A	110.4
C3—C2—C1	120.1 (4)	S1—C18—H18A	110.4
C3—C2—H2	119.9	N1—C18—H18B	110.4
C1—C2—H2	119.9	S1—C18—H18B	110.4
C2—C3—C4	119.9 (4)	H18A—C18—H18B	108.6
C2—C3—H3	120.1	N1—C19—C33	117.75 (17)
C4—C3—H3	120.1	N1—C19—C20	102.15 (16)
C3—C4—C5	120.3 (4)	C33—C19—C20	114.44 (16)
C3—C4—H4	119.9	N1—C19—C24	107.62 (16)
C5—C4—H4	119.9	C33—C19—C24	102.38 (16)
C6—C5—C4	120.5 (3)	C20—C19—C24	112.68 (17)
C6—C5—H5	119.8	C21—C20—C15	114.78 (18)
C4—C5—H5	119.8	C21—C20—C19	113.89 (17)
C5—C6—C1	117.9 (3)	C15—C20—C19	103.72 (16)
C5—C6—C7	121.5 (3)	C21—C20—H20	108.0
C1—C6—C7	120.5 (3)	C15—C20—H20	108.0
O4—C7—C6	110.0 (2)	C19—C20—H20	108.0
O4—C7—H7A	109.7	O1—C21—O2	124.9 (2)
C6—C7—H7A	109.7	O1—C21—C20	124.2 (2)
O4—C7—H7B	109.7	O2—C21—C20	110.9 (2)
C6—C7—H7B	109.7	O3—C24—C25	128.6 (2)
H7A—C7—H7B	108.2	O3—C24—C19	123.6 (2)
O4—C8—C9	110.44 (18)	C25—C24—C19	107.87 (18)
O4—C8—C12	109.82 (16)	C26—C25—C34	119.6 (2)
C9—C8—C12	100.97 (16)	C26—C25—C24	132.6 (2)
O4—C8—H8	111.7	C34—C25—C24	107.80 (19)
C9—C8—H8	111.7	C25—C26—C27	118.0 (3)
C12—C8—H8	111.7	C25—C26—H26	121.0
O7—C9—C8	108.83 (19)	C27—C26—H26	121.0
O7—C9—C11	103.87 (17)	C28—C27—C26	122.4 (3)
C8—C9—C11	103.81 (17)	C28—C27—H27	118.8
O7—C9—H9	113.2	C26—C27—H27	118.8
C8—C9—H9	113.2	C27—C28—C29	121.1 (2)
C11—C9—H9	113.2	C27—C28—H28	119.5
O6—C10—O7	105.64 (17)	C29—C28—H28	119.5
O6—C10—C14	109.8 (3)	C34—C29—C28	115.6 (2)
O7—C10—C14	108.7 (3)	C34—C29—C30	116.7 (2)
O6—C10—C13	110.0 (2)	C28—C29—C30	127.6 (2)
O7—C10—C13	109.6 (2)	C31—C30—C29	119.8 (2)
C14—C10—C13	112.9 (3)	C31—C30—H30	120.1
O6—C11—O5	111.7 (2)	C29—C30—H30	120.1
O6—C11—C9	104.83 (17)	C30—C31—C32	122.5 (2)
O5—C11—C9	106.55 (16)	C30—C31—H31	118.7
O6—C11—H11	111.2	C32—C31—H31	118.7
O5—C11—H11	111.2	C33—C32—C31	119.4 (2)
C9—C11—H11	111.2	C33—C32—H32	120.3
O5—C12—C8	102.65 (17)	C31—C32—H32	120.3
O5—C12—C15	111.29 (15)	C32—C33—C34	118.1 (2)
C8—C12—C15	116.53 (17)	C32—C33—C19	133.6 (2)
O5—C12—H12	108.7	C34—C33—C19	108.30 (18)
C8—C12—H12	108.7	C29—C34—C25	123.2 (2)
C15—C12—H12	108.7	C29—C34—C33	123.4 (2)
C10—C13—H13A	109.5	C25—C34—C33	113.27 (19)
C10—C13—H13B	109.5	C18—N1—C19	118.65 (18)
H13A—C13—H13B	109.5	C18—N1—C16	111.09 (18)
C10—C13—H13C	109.5	C19—N1—C16	111.32 (15)
H13A—C13—H13C	109.5	C7—O4—C8	113.87 (18)
H13B—C13—H13C	109.5	C11—O5—C12	108.05 (15)
C10—C14—H14A	109.5	C11—O6—C10	111.37 (16)
C10—C14—H14B	109.5	C9—O7—C10	109.72 (17)
H14A—C14—H14B	109.5	C17—S1—C18	87.71 (11)
C10—C14—H14C	109.5	C21—O2—C22'	125.8 (7)
H14A—C14—H14C	109.5	C21—O2—C22	110.4 (4)
H14B—C14—H14C	109.5	O2—C22'—C23'	101.0 (8)
C20—C15—C16	102.45 (16)	O2—C22'—H22A	111.6
C20—C15—C12	114.38 (17)	C23'—C22'—H22A	111.6
C16—C15—C12	115.66 (16)	O2—C22'—H22B	111.6
C20—C15—H15	108.0	C23'—C22'—H22B	111.6
C16—C15—H15	108.0	H22A—C22'—H22B	109.4
C12—C15—H15	108.0	C23—C22—O2	107.0 (7)
N1—C16—C17	109.70 (16)	C23—C22—H22C	110.3
N1—C16—C15	104.77 (16)	O2—C22—H22C	110.3
C17—C16—C15	114.75 (18)	C23—C22—H22D	110.3
N1—C16—H16	109.1	O2—C22—H22D	110.3
C17—C16—H16	109.1	H22C—C22—H22D	108.6
C15—C16—H16	109.1	C22—C23—H23D	109.5
C16—C17—S1	106.38 (15)	C22—C23—H23E	109.5
C16—C17—H17A	110.5	H23D—C23—H23E	109.5
S1—C17—H17A	110.5	C22—C23—H23F	109.5
C16—C17—H17B	110.5	H23D—C23—H23F	109.5
S1—C17—H17B	110.5	H23E—C23—H23F	109.5
			
C6—C1—C2—C3	−3.2 (7)	C28—C29—C30—C31	177.8 (3)
C1—C2—C3—C4	−0.6 (8)	C29—C30—C31—C32	1.8 (4)
C2—C3—C4—C5	2.2 (8)	C30—C31—C32—C33	−0.3 (4)
C3—C4—C5—C6	0.0 (7)	C31—C32—C33—C34	−1.2 (3)
C4—C5—C6—C1	−3.6 (5)	C31—C32—C33—C19	−178.8 (2)
C4—C5—C6—C7	180.0 (4)	N1—C19—C33—C32	−69.6 (3)
C2—C1—C6—C5	5.3 (6)	C20—C19—C33—C32	50.4 (3)
C2—C1—C6—C7	−178.3 (4)	C24—C19—C33—C32	172.7 (2)
C5—C6—C7—O4	−131.1 (3)	N1—C19—C33—C34	112.6 (2)
C1—C6—C7—O4	52.6 (4)	C20—C19—C33—C34	−127.35 (19)
O4—C8—C9—O7	163.47 (15)	C24—C19—C33—C34	−5.1 (2)
C12—C8—C9—O7	−80.38 (19)	C28—C29—C34—C25	−2.6 (3)
O4—C8—C9—C11	−86.4 (2)	C30—C29—C34—C25	176.9 (2)
C12—C8—C9—C11	29.8 (2)	C28—C29—C34—C33	−179.3 (2)
O7—C9—C11—O6	−12.6 (2)	C30—C29—C34—C33	0.1 (3)
C8—C9—C11—O6	−126.3 (2)	C26—C25—C34—C29	2.4 (4)
O7—C9—C11—O5	105.99 (19)	C24—C25—C34—C29	−175.3 (2)
C8—C9—C11—O5	−7.8 (2)	C26—C25—C34—C33	179.4 (2)
O4—C8—C12—O5	74.99 (18)	C24—C25—C34—C33	1.7 (3)
C9—C8—C12—O5	−41.62 (19)	C32—C33—C34—C29	1.3 (3)
O4—C8—C12—C15	−46.9 (2)	C19—C33—C34—C29	179.4 (2)
C9—C8—C12—C15	−163.49 (18)	C32—C33—C34—C25	−175.8 (2)
O5—C12—C15—C20	128.42 (18)	C19—C33—C34—C25	2.4 (3)
C8—C12—C15—C20	−114.4 (2)	S1—C18—N1—C19	100.49 (19)
O5—C12—C15—C16	9.7 (3)	S1—C18—N1—C16	−30.4 (2)
C8—C12—C15—C16	126.9 (2)	C33—C19—N1—C18	−21.1 (3)
C20—C15—C16—N1	29.11 (19)	C20—C19—N1—C18	−147.37 (18)
C12—C15—C16—N1	154.21 (17)	C24—C19—N1—C18	93.8 (2)
C20—C15—C16—C17	149.47 (18)	C33—C19—N1—C16	109.69 (19)
C12—C15—C16—C17	−85.4 (2)	C20—C19—N1—C16	−16.6 (2)
N1—C16—C17—S1	25.76 (19)	C24—C19—N1—C16	−135.40 (17)
C15—C16—C17—S1	−91.84 (18)	C17—C16—N1—C18	3.3 (2)
C16—C15—C20—C21	−164.34 (17)	C15—C16—N1—C18	126.92 (18)
C12—C15—C20—C21	69.7 (2)	C17—C16—N1—C19	−131.35 (17)
C16—C15—C20—C19	−39.46 (19)	C15—C16—N1—C19	−7.7 (2)
C12—C15—C20—C19	−165.40 (15)	C6—C7—O4—C8	−178.8 (2)
N1—C19—C20—C21	160.00 (19)	C9—C8—O4—C7	−113.4 (2)
C33—C19—C20—C21	31.6 (3)	C12—C8—O4—C7	136.1 (2)
C24—C19—C20—C21	−84.8 (2)	O6—C11—O5—C12	94.59 (19)
N1—C19—C20—C15	34.54 (19)	C9—C11—O5—C12	−19.3 (2)
C33—C19—C20—C15	−93.84 (19)	C8—C12—O5—C11	38.9 (2)
C24—C19—C20—C15	149.72 (17)	C15—C12—O5—C11	164.21 (18)
C15—C20—C21—O1	−4.0 (3)	O5—C11—O6—C10	−115.1 (2)
C19—C20—C21—O1	−123.3 (3)	C9—C11—O6—C10	−0.1 (3)
C15—C20—C21—O2	175.35 (19)	O7—C10—O6—C11	12.7 (3)
C19—C20—C21—O2	56.0 (3)	C14—C10—O6—C11	129.8 (3)
N1—C19—C24—O3	61.5 (3)	C13—C10—O6—C11	−105.4 (3)
C33—C19—C24—O3	−173.8 (2)	C8—C9—O7—C10	131.1 (2)
C20—C19—C24—O3	−50.3 (3)	C11—C9—O7—C10	21.0 (2)
N1—C19—C24—C25	−118.61 (18)	O6—C10—O7—C9	−21.5 (3)
C33—C19—C24—C25	6.1 (2)	C14—C10—O7—C9	−139.2 (2)
C20—C19—C24—C25	129.55 (18)	C13—C10—O7—C9	97.0 (3)
O3—C24—C25—C26	−2.4 (4)	C16—C17—S1—C18	−36.36 (15)
C19—C24—C25—C26	177.7 (3)	N1—C18—S1—C17	38.98 (17)
O3—C24—C25—C34	174.8 (2)	O1—C21—O2—C22'	−11.9 (7)
C19—C24—C25—C34	−5.0 (2)	C20—C21—O2—C22'	168.8 (6)
C34—C25—C26—C27	−0.5 (4)	O1—C21—O2—C22	5.5 (6)
C24—C25—C26—C27	176.5 (3)	C20—C21—O2—C22	−173.8 (6)
C25—C26—C27—C28	−0.9 (5)	C21—O2—C22'—C23'	97.2 (11)
C26—C27—C28—C29	0.7 (5)	C22—O2—C22'—C23'	47.9 (17)
C27—C28—C29—C34	1.1 (4)	C21—O2—C22—C23	159.1 (8)
C27—C28—C29—C30	−178.4 (3)	C22'—O2—C22—C23	−62 (2)
C34—C29—C30—C31	−1.6 (4)		

### Hydrogen-bond geometry (Å, º)

**Table:**

*D*—H···*A*	*D*—H	H···*A*	*D*···*A*	*D*—H···*A*
C5—H5···O3^i^	0.93	2.51	3.375 (4)	155
C17—H17*A*···O7^ii^	0.97	2.44	3.309 (3)	148
C23—H23*F*···O4^iii^	0.96	2.57	3.497 (9)	163
C31—H31···O5^iv^	0.93	2.47	3.393 (4)	173

Symmetry codes: (i) −*x*+1, *y*+1/2, −*z*; (ii) *x*+1, *y*, *z*; (iii) −*x*+1, *y*−1/2, −*z*; (iv) *x*, *y*, *z*−1.
